# Development of Novel Protocol to Al^3+^ Stress Tolerance at Germination Stage in* Indica* Rice through Statistical Approaches

**DOI:** 10.1155/2018/8180174

**Published:** 2018-09-26

**Authors:** Nusrat Jahan, Fazilah Abd Manan, Arsala Mansoor, Mudassir Asrar Zaidi, Muhammad Naeem Shahwani, Muhammad Arshad Javed

**Affiliations:** ^1^Department of Biotechnology, Faculty of Life Sciences & Informatics, Balochistan University of Information Technology, Engineering & Management Sciences, Quetta, Pakistan; ^2^Faculty of Biosciences and Medical Engineering, Universiti Teknologi Malaysia, 81310 Johor Bahru, Malaysia; ^3^Faculty of Natural Sciences, University of Mississippi, MS 38677, USA; ^4^Department of Botany, University of Balochistan, Pakistan

## Abstract

Rice production is decreasing by abiotic stresses like heavy metals. In such circumstances, producing food for growing human population is a challenge for plant breeders. Excess of Al^3+^ in soil has become threat for high yield of rice. Improvement of crop is one of potential solution for high production. The aim of this study was to develop the new method for optimization of Al^3+^ toxicity tolerance in* indica *rice at germination stag using two-way ANOVA and Duncan's multiple-range test (DMRT). Seeds of two* indica* rice cultivars (*Pokkali* and* Pak Basmati)* were exposed in different concentrations (control, 5 mM, 15 mM, and 20 mM) of Al^3+^ toxicity at pH 4 ±0.2 for two weeks. Germination traits such as final germination percentage (FG%), germination energy (GE), germination speed (GS), germination index (GI), mean time of germination (MGT), germination value (GV), germination velocity (GVe), peak value of germination (GPV), and germination capacity (GC) and growth traits such as root length (RL), shoot length (SL), total dry biomass (TDB), and germination vigour index (GVI) were measured. To obtain the maximum number of significance (≤ 0.01%) parameters in each concentration of Al^3+^ toxicity with control, two-way ANOVA was established and comparison of mean was done using DMRT. The results showed that 5 mM, 10 mM, and 15 mM have less significant effects on the above-mentioned parameters. However, 20 mM concentration of Al^3+^ produced significant effects (≤ 0.01%). Therefore, 20 mM of Al^3+^ is considered optimized limit for* indica* cultivars (*Pokkali* and* Pak Basmati*).

## 1. Background

Acidic soils are one of the main constraints for crop production. Almost 30-40% of world soils have a pH below 5.5 [1]. The lower the pH, the more acidic the soil. Acidic soils are low in fertility due to the presence of combined mineral toxicities (Al^3+^, Mn^2+^, and Fe^2+^) and deficiency of macronutrients (phosphorous (P), calcium (Ca), and magnesium (Mg) [2]. At low pH of the soil, aluminum and other various species like Fe^2+^ and Mn^2+^ are solubilized into the soil, which are severely toxic to rice crop production. Heavy rainfall and high-temperature cause the rapid weathering of soil and the essential elements like Ca, P, and K leach from the soil; more stable compounds rich in Al^3+^ and Fe^2+^ oxides are left behind [3]. Al^3+^ is primarily found as a significant component of soil clays. Under highly acidic soil conditions (pH<5.0) it is solubilized to Al^3+^, which is highly phytotoxic. Al^3+^ affects the root growth rapidly that causes the reduced and stunted root system and has a direct effect on the ability of a crop to acquire both water and nutrients.

Al^3+^ toxicity is reducing production on acidic soils due to inhibition of root growth, reduction in cell division, and cell elongation [4]. To reduce the cell elongation, Al^3+^ may bind to free carboxyl groups of pectin, resulting in cross-linking of pectin molecules and a decrease in cell wall elasticity [5].

Acidic soils are becoming an issue with the changing environment; reduction of available arable lands due to weathering of soils, unsustainable farming and toxic soils, rigorous agricultural practices, acid rain, and climate change are the contributors to soil acidification [6, 7]. Cultural strategies, like application of lime (CaCO_3_), could amend the few constraints of acidic soils and lead to increase in production [6]. However, liming is only effective at increasing the pH in the upper soil profile and is mostly unproductive when the subsoil is acidic [8]. It has reported that approximately 75% of the acidic soils in the world are influenced by subsoil acidity. In many regions of the world, liming is also not effective due to high cost and lacking of infrastructure. Therefore, developing Al^3+^ tolerant crops tolerating the acidic soils has great importance for breeding programs worldwide. Identification of QTL linked to tolerance traits is one of the important techniques. The aim of present study was to find the statistical approach that could ease for optimization of Al^3+^ toxicity tolerance level for two commonly used* indica* rice* Pokkali *and* Pak Basmati* against high concentrations of Al^3+^ toxicity at germination stage.

## 2. Materials and Methods

The parental genotypes,* Pak Basmati* and* Pokkali,* were exposed to different levels of Al^3+^ toxicity to optimize the optimum stress limits. Seeds were kept in 50°C for five days to break dormancy and surface sterilized by dipping in 70% (v/v) ethanol for 1 min and in 2% (w/v) solution of NaOCl for 10min followed by washing 4-5 times with deionized water [9]. Surface sterilized and imbedded seeds were then placed in wet Petri dishes for two weeks by the addition of Al^3+^ stresses (control, 5 mM,15 mM, and 20 mM) at pH4.0-4.2; each treatment had three replications where it has been determined to be a good standardization to natural soil condition where Al^3+^ toxicity is the problem [10]. Experiments were conducted in control condition, where the light and dark periods were 14 hours and 10 hours, respectively, with humidity level of approx. 60%. Seeds were considered germinated when both the plumule (root) and radical (shoot) were extended to approximately more than 2mm [11]. Germination parameters such as final germination percentage (FG %), germination velocity (GVe), germination energy (GE), germination peak value (GPV), germination capacity (GC), germination index (GI), germination value (GV) and growth parameters like root length (RL), shoot length (SL), total dry biomass (TDB), and germination vigour index (GVI) were recorded by the following formulas.

(1) i  FG%=Number  of  germinated  seedsTotal  number  of  seeds  testedii  GVe=∑Gt

where G is germination percentage and t is total germination time;

(2) iii  MGT=ΣDnΣn  daysiv  GE%=Number  of  germinated  seeds  at  4  DASTotal  number  of  seeds  tested×100v  GV=finalMDG×PVvi  SG=Number  of  germinated  seedsdays  of  1st  count+⋯+Number  of  germinated  seedsdays  of  final  count  9  daysvii  GPV=Cumulative  Percent  Germination  on  each  daynumber  of  days  after  germination  elapsed.viii  GI=N10+N1520×100

where N10 is number of germinated seeds with 10 days of stress and N15 is number of germinated seeds with 15 days of the stress

(3) xi  GC=Percentage  of  seeds  germinated  at  160  hours.x  GVI=Avg  shoot  length+Avg  root  length×Germination  percentage

[12–14].

### 2.1. Statistical Analysis

Statistical analysis was done with SPSS version 18 (Levesque, 2007). To establish the different significance of variables in each concentration of Al^3+^ toxicity with control, analysis of variance (two-way ANOVA) was tested. Two significance levels, p (≤0.05 to ≤0.01), were used [15]. Differences between genotypes were compared using Duncan's multiple-range test (DMRT). Al^3+^ concentration was considered as optimized where most of germination and growth parameters exhibited high significant differences [16]

## 3. Results and Discussion

The inhibitory effects of Al^3+^ toxicity were checked on rice genotypes* Pak Basmati* and* Pokkali*, germination and seedling growth parameters were examined over a wide range of AlCl_3_ from 5 mM to 20 mM with three replications. ANOVA was applied to the germination and growth parameters of all treatments, i.e., 5 mM, 10 mM, 15 mM, and 20 mM.

Analysis of variance showed that the germination parameters in 5 mM of AlCl_3_ are relatively less sensitive in both* Pak Basmati* and* Pokkali* as shown in Table 1. No significant variations were observed in germination parameters while high significant (p≤0.01) difference in seedling growth parameters was observed. However, ANOVA results showed the difference in germination parameters between 5 mM and 10 mM of AlCl_3_ that were relatively small sensitivity in both* indica* cultivars* Pak Basmati* and* Pokkali*. Al^3+^ toxicity treatments at 15 mM and 20 mM produced significant (p≤0.01) effects on all germination and seedling growth parameters except in final germination percentage, germination velocity, and germination index (GI) as shown in Tables 1 and 2.

Comparison of mean showed that, with increasing levels in Al^+3^ toxicity, there was a reduction in germination and seedling growth parameters as presented in Table 3. A significant influence of Al^3+^ toxicity was observed in 15 mM and 20 mM, while the least effect was found out in 5 mM and 10 mM showing that these genotypes are Al^3+^ tolerant varieties.

The germination parameters and seedling growth parameters in 10 mM of AlCl_3_ were more affected relative to 5 mM; however, at 10 mM concentration of Al^3+^ produced less number of significant effects (p<0.01) on germination traits in all source of variables (Tables 1 and 2) which reflects that rice genotypes were responding the same in 10 mM of Al^3+^. The difference in the results of all germination and growth parameters of both varieties between 15 mM and 20 mM was germination index (GI) producing strong significant (p<0.01) variation in 20 mM while in 15 mM it was significant at 0.05%; similarly mean time of germination (MGT) was significant (p<0.05) for factor variety and highly significant (p<0.01) for stress at 20 mM of Al^3+^ toxicity but it was significant (p<0.05) for factor variety only at 15 mM of Al^3+^ toxicity. Similarly, germination capacity was significant (p<0.05) for all factors in 15 mM while it was highly significant (p<0.01) for stress under 20 mM Al^3+^ toxicity. Similar kind of response has been reported by Nasr [17] while investigating the germination and seedling growth of maize (*Zea mays* L.) seeds in toxicity of aluminum and nickel that Al^3+^ treatments significantly (p<0.05) decreased seed germination as compared to control and 2000 mg/L (20 mM) showed the lowest percentage of tolerance in maize seedlings as compared to control. The reduction in seed germination of maize* (Zea mays* L.) can be due to the accelerated breakdown of stored food material in seed by the application of Al^3+^ [18]. Consequently, the concentration 20 mM of Al^3+^ toxicity was selected as a threshold for phenotyping in QTL analysis [5], since its results showed the maximum significance (p<0.01) in germination and seedling growth parameters.

## 4. Conclusion

The genotypes* Pokkali* and* Pak Basmati* showed significance difference (p<0.01) when exposed to optimized concentration, i.e., 20 mM (2000mg/L). The genotype* Pokkali* showed stronger tolerance than the* Pak Basmati* in all parameters, especially in root length. Al^3+^ concentration is considered as optimized where most of germination and growth parameters exhibited high significant differences. In addition, promising statistical approaches for optimization of toxicity limits are being developed for phenotyping of population and identifying QTLs that could be used in crop improvement.

## Figures and Tables

**Table 1:** Analysis of variance of 5 mM and 10 mM of Al^3+^ toxicity at pH4 ±0.2 in germination parameters on parental line *Pak Basmati* and *Pokkali.*

**Source of variations**	**df**	**Sum of Square**												
		**FG**%	**GVe**	**GE**	**SG**	**GPV**	**GI**	**GC**	**GV**	**MGT**	**RL**	**SL**	**TDB**	**VI**
variety	1	0.00^ns^	0.00^ns^	208.33^ns^	17.45^*∗∗*^	3.467^*∗∗*^	0.00^ns^	15.64^*∗∗*^	0.00^ns^	154.15^*∗∗*^	0.03^*∗∗*^	4.663^*∗∗*^	23.46^*∗∗*^	23.46^*∗∗*^

Stress	1	0.00^ns^	0.00^ns^	75.00^ns^	0.13^ns^	0.02^ns^	0.00^ns^	0.141	0.00^ns^	1.21^ns^	0.00^*∗∗*^	24.027^*∗∗*^	1.48^*∗∗*^	1.48^*∗∗*^

variety × Stress	1	0.00^ns^	0.00^ns^	75.00^ns^	7.50^ns^	0.00^ns^	0.00^ns^	0.007	0.00^ns^	0.00^ns^	0.00^*∗∗*^	2.94^*∗∗*^	0.15^*∗∗*^	0.15^*∗∗*^

Error	8	0.00^ns^	0.00	333.33	0.82	0.165	0.00	0.54	0.00	7.21	0.00	0.01	0.027	0.027

Total	12													

variety	1	0.00^ns^	0.00^ns^	208.33^ns^	16.97^*∗∗*^	3.35^*∗∗*^	0.00^ns^	15.64^*∗∗*^	0.00^ns^	149.11^*∗∗*^	0.026^*∗∗*^	23.21^*∗∗*^	23.21^*∗∗*^	457470.75^*∗∗*^

Stress	1	0.00^ns^	0.00^ns^	75.00^ns^	0.161^ns^	0.03^ns^	0.00^ns^	0.14^ns^	0.00^ns^	1.38^ns^	0.007^*∗∗*^	1.76^*∗∗*^	1.77^*∗∗*^	414780.08^*∗∗*^

variety × Stress	1	0.00^ns^	0.00^ns^	75.00^ns^	0.00^ns^	0.00^ns^	0.00^ns^	0.00^ns^	0.00^ns^	0.05^ns^	0.001^*∗∗*^	0.13^*∗∗*^	0.134^*∗∗*^	24570.75^*∗∗*^

Error	8	0.00	0.00	333.33	0.27^ns^	0.05^ns^	0.00	0.54	0.00	2.437	0.00	0.038	0.038	722.667

Total	12													

^*∗∗*^Significant at 0.01, *∗* significant at 0.05, ns= not significant, FG%= final germination percentage, GVe = germination velocity, GE= germination energy, SG=speed of germination, GPV= germination peak value, GI=germination index, GC= germination capacity, GV=germination value, MGT=mean germination time, RL=root length, SL = shoot length, TDB= total dry biomass, and GVI= germination vigour index.

**Table 2:** Analysis of variance of 15 mM and 20 mM of Al^3+^ toxicity at pH4 ±0.2 in germination parameters on parental line *Pak Basmati* and *Pokkali.*

**Source of variations**	**df**	**Sum of Square**												
		**FG**%	**GVe**	**GE**	**SG**	**GPV**	**GI**	**GC**	**GV**	**MGT**	**RL**	**SL**	**TDB**	**VI**
variety	1	33.33^ns^	0.15^ns^	1200^*∗∗*^	27.36^*∗∗*^	6.44^*∗∗*^	133.33^*∗*^	71.74^*∗*^	133.33^*∗*^	371.85^*∗∗*^	0.028^*∗∗*^	3.842^*∗∗*^	24.74^*∗∗*^	514022.41^*∗∗*^

Stress	1	33.33^ns^	0.15^ns^	833.33^*∗∗*^	2.90^*∗∗*^	0.94^*∗∗*^	133.33^*∗*^	25.64^ns^	133.33^*∗*^	77.72	0.01^*∗∗*^	27.09^*∗∗*^	2.314^*∗∗*^	483847.68^*∗∗*^

variety × Stress	1	33.33^ns^	0.15^ns^	833.33^*∗∗*^	1.07^*∗∗*^	0.45^*∗∗*^	133.33^*∗*^	21.17^ns^	133.33^*∗*^	46.65	0.001^*∗*^	3.663^*∗∗*^	0.27^*∗∗*^	13493.81^*∗∗*^

Error	8	66.67	0.30	133.33	0.411	0.057	66.67	28.27	66.667	14.452	0	0.008	0.023	1615.74

Total	12													

variety	1	75.00^ns^	0.34^ns^	833.33^*∗∗*^	36.79^*∗∗*^	7.29^*∗∗*^	675.00^*∗∗*^	152.44^*∗*^	408.33	366.86^*∗∗*^	0.03^*∗∗*^	3.95^*∗∗*^	13.29^*∗∗*^	311793.04^*∗∗*^

Stress	1	675.00^ns^	3.03^ns^	7500^*∗∗*^	98.09^*∗∗*^	19.38^*∗∗*^	3675.00^*∗∗*^	308.74^*∗∗*^	3008.33^*∗∗*^	1409.418^*∗∗*^	0.02^*∗∗*^	32.28^*∗∗*^	55.17^*∗∗*^	1833399.19^*∗∗*^

variety × Stress	1	75.00^ns^	0.34^ns^	533.33^*∗∗*^	3.49^*∗∗*^	0.70^*∗∗*^	675.00^*∗∗*^	71.88^ns^	408.33^ns^	44.89^*∗∗*^	0.00^*∗∗*^	3.56^*∗∗*^	0.65^*∗∗*^	75477.74^*∗∗*^

Error	8	0.00	0.00	200.00	1.51	0.30	200.00	65.44	266.67	9.76	0.00	0.01	0.32	2299.46

Total	12													

^*∗∗*^Significant at 0.01, *∗* significant at 0.05, ns= not significant FG%= final germination percentage, GVe = germination velocity, GE= germination energy, SG=speed of germination, GPV= germination peak value, GI=germination index, GC= germination capacity, GV=germination value, MGT=mean germination time, RL=root length, SL = shoot length, TDB= total dry biomass, and GVI= germination vigour index.

**Table 3:** Comparison of mean using DMRT for effect of Al^3+^ on germination and growth traits of rice genotypes.

**Varieties**	**Treatments**	**FGP**	**GVe**	**GEn**	**SG**	**GPV**	**GC**	**GI**	**MGT**	**GV**	**TDW**	**RL**	**SL**	**GVI**
*Pokkali*	control	100±0.0a	6.67±0.0a	100±0.0a	18.34±0.2a	8.15±0.16a	100±0.0a	200±0.0a	117.06±0.1a	54.36±0.8a	0.28±0.0a	4.43±0.0a	7.49±0.0a	1192.66±0.0a
	5 mM	100±0.0a	6.67±0.0a	100±0.0a	18.07±0.1a	8.03±0.08a	100±0.0a	200±0.0a	116.90±0.1ab	53.54±0.5ab	0.21±0.0ab	0.36±0.0b	6.94±0.0ab	730.33±0.0b
	10 mM	100±0.0a	6.67±0.3a	100±0.0a	17.96±0.1ab	7.98±0.08ab	100±0.0a	200±0.0a	116.80±0.1ab	53.21±0.5ab	0.21±0.0ab	0.32±0.0bc	6.91±0.0b	724.00±0.0bc
	15 mM	93.33±0.5ab	6.62±0.3ab	66.66±0.5b	14.45±0.1b	6.42±0.07c	90.00±0.0ab	186.66±0.1ab	112.58±0.2b	40.00±0.9b	0.21±0.0ab	0.11±0.0c	5.79±0.0bc	550.56±0.3c
	20 mM	86.66±0.5b	5.77±0.4b	53.33±0.5c	12.15±0.9c	5.40±0.39d	76.66±0.1b	173.33±0.7b	951.66±0.5c	31.24±0.6c	0.18±0.0b	0.06±0.0cd	2.74±0.0c	243.03±0.1cd

*Pak Basmati*	Control	100±0.0a	6.67±0.0a	100±0.0a	15.92±0.1a	7.07±0.04a	100±0.0a	200±0.0a	114.83±0.3a	47.17±0.2a	0.17±0.0a	2.19±0.01a	4.92±0.1a	711.66±0.0a
	5 mM	100±0.01a	6.67±0.0a	96.66±0.3ab	15.73±0.0a	6.99±0.04ab	100±0.0a	200±0.0a	114.56±0.3ab	46.63±0.2ab	0.14±0.0ab	0.35±0.0b	3.96±0.0ab	430.33±0.0b
	10 mM	93.33±0.0ab	6.62±0.3ab	86.66±0.3b	14.34±0.2ab	6.44±0.05ab	90±0.5ab	186.66±0.5ab	112.90±0.3b	40.14±0.4bc	0.14±0.0ab	0.32±0.0bc	3.76±0.0b	377.13±0.0bc
	15 mM	93.33±0.6ab	6.62±0.3ab	70.00±0.4bc	14.15±0.2ab	6.13±0.24bc	86.66±0.0b	186.66±0.5ab	109.52±0.2bc	38.14±0.6c	0.13±0.0b	0.08±0.0c	3.26±0.0bc	310.16±0.2c
	20 mM	70.66±0.5c	5.70±0.1b	63.33±0.4c	13.76±0.1c	5.90±0.31c	70.00±0.0c	164.43±0.7b	346.86±1.0c	30.07±0.7cd	0.12±0.8bc	0.06±0.0cd	2.59±0.1c	64.04±0.4d

*∗*Same letters are not significantly different at probability (p<5%) error by Duncan's multiple test. Values are means ± SD=standard deviation FG%= final germination percentage, GVe = germination velocity, GE= germination energy, SG=speed of germination, GPV= germination peak value, GI=germination index, GC= germination capacity, GV=germination value, MGT=mean germination time, RL=root length, SL = shoot length, TDB= total dry biomass, and GVI= germination vigour index.

## Data Availability

The data used to support the findings of this study are available from the corresponding author upon request.

## Abbreviations

**QTL**: Quantitative trait loci
mM: Millimole
mg: Milligram
L: Litre.
